# LiKidMiRs: A ddPCR-Based Panel of 4 Circulating miRNAs for Detection of Renal Cell Carcinoma

**DOI:** 10.3390/cancers14040858

**Published:** 2022-02-09

**Authors:** José Pedro Sequeira, Vera Constâncio, Sofia Salta, João Lobo, Daniela Barros-Silva, Carina Carvalho-Maia, Jéssica Rodrigues, Isaac Braga, Rui Henrique, Carmen Jerónimo

**Affiliations:** 1 Cancer Biology and Epigenetics Group, Research Center of IPO Porto (CI-IPOP)/RISE@CI-IPOP (Health Research Network), Portuguese Oncology Institute of Porto (IPO Porto)/Porto Comprehensive Cancer Centre (Porto.CCC), R. Dr. António Bernardino de Almeida, 4200-072 Porto, Portugal; jose.leite.sequeira@ipoporto.min-saude.pt (J.P.S.); vera.salvado.constancio@ipoporto.min-saude.pt (V.C.); sofia.salta@ipoporto.min-saude.pt (S.S.); jpedro.lobo@ipoporto.min-saude.pt (J.L.); daniela.silva@ipoporto.min-saude.pt (D.B.-S.); carina.carvalho.maia@ipoporto.min-saude.pt (C.C.-M.); 2 Master Programme in Oncology, School of Medicine & Biomedical Sciences, University of Porto (ICBAS-UP), Rua Jorge Viterbo Ferreira 228, 4050-513 Porto, Portugal; 3 Doctoral Programme in Biomedical Sciences, School of Medicine & Biomedical Sciences, University of Porto (ICBAS-UP), Rua Jorge Viterbo Ferreira 228, 4050-513 Porto, Portugal; 4 Doctoral Programme in Molecular Pathology and Genetics, School of Medicine & Biomedical Sciences, University of Porto (ICBAS-UP), Rua Jorge Viterbo Ferreira 228, 4050-513 Porto, Portugal; 5 Department of Pathology, Portuguese Oncology Institute of Porto (IPOP), R. Dr. António Bernardino de Almeida, 4200-072 Porto, Portugal; 6 Department of Pathology and Molecular Immunology, Institute of Biomedical Sciences Abel Salazar, University of Porto (ICBAS-UP), Rua Jorge Viterbo Ferreira 228, 4050-513 Porto, Portugal; 7 Cancer Epidemiology Group, IPO Porto Research Center of IPO Porto (CI-IPOP)/RISE@CI-IPOP (Health Research Network), Portuguese Oncology Institute of Porto (IPO Porto)/Porto Comprehensive Cancer Centre (Porto.CCC), R. Dr. António Bernardino de Almeida, 4200-072 Porto, Portugal; jessica.rocha.rodrigues@ipoporto.min-saude.pt; 8 Centre of Mathematics (CMAT), University of Minho, Campus de Gualtar, R. da Universidade, 4710-057 Braga, Portugal; 9 Department of Urology & Urology Clinics, Portuguese Oncology Institute of Porto (IPOP), R. Dr. António Bernardino de Almeida, 4200-072 Porto, Portugal; isaac.braga@ipoporto.min-saude.pt

**Keywords:** ddPCR, circulating miRNA, renal cell carcinoma, diagnosis, malignancy

## Abstract

**Simple Summary:**

Early detection of renal cell carcinoma (RCC) significantly increases the likelihood of curative treatment, avoiding the need of adjuvant therapies, associated side effects and comorbidities. Thus, we aimed to discover circulating microRNAs that might aid in early, minimally invasive, RCC detection/diagnosis.

**Abstract:**

Background: Decreased renal cell cancer-related mortality is an important societal goal, embodied by efforts to develop effective biomarkers enabling early detection and increasing the likelihood of curative treatment. Herein, we sought to develop a new biomarker for early and minimally invasive detection of renal cell carcinoma (RCC) based on a microRNA panel assessed by ddPCR. Methods: Plasma samples from patients with RCC (*n* = 124) or oncocytomas (*n* = 15), and 64 healthy donors, were selected. Hsa-miR-21-5p, hsa-miR-126-3p, hsa-miR-155-5p and hsa-miR-200b-3p levels were evaluated using a ddPCR protocol. Results: RCC patients disclosed significantly higher circulating levels of hsa-miR-155-5p compared to healthy donors, whereas the opposite was observed for hsa-miR-21-5p levels. Furthermore, hsa-miR-21-5p and hsa-miR-155-5p panels detected RCC with high sensitivity (82.66%) and accuracy (71.89%). The hsa-miR-126-3p/hsa-miR-200b-3p panel identified the most common RCC subtype (clear cell, ccRCC) with 74.78% sensitivity. Conclusion: Variable combinations of plasma miR levels assessed by ddPCR enable accurate detection of RCC in general, and of ccRCC. These findings, if confirmed in larger studies, provide evidence for a novel ancillary tool which might aid in early detection of RCC.

## 1. Introduction

Renal cancer remains one of the leading urologic cancers worldwide, being listed as one of the twenty most common and deadly cancers, especially among men (1.5:1) [1,2].

Renal cell tumors (RCTs) correspond to a set of benign and malignant neoplasms, with extensive diversity at epigenetic, molecular, and clinical levels [3,4]. Among them, about 10% correspond to benign tumors, with oncocytomas constituting the most common benign tumor [1,4]. Concerning malignant RCTs, clear-cell renal cell carcinoma (ccRCC) is the most common subtype (65–75% of all RCCs) [5], followed by papillary renal cell carcinomas (pRCC, ~16%) and chromophobe renal cell carcinomas (chRCC, ~7%)[5]. RCCs derive from nephron epithelial cells [1,6,7] and are characterized by their heterogeneity, both morphological and molecular. Whereas localized RCC is mostly cured by surgery, locally advanced or systemic disease constitute major therapeutic challenges, entailing the need for development not only of biomarkers for early detection, but also novel therapies [8].

In recent years, several studies have been published concerning the use of circulating microRNAs (miRNAs) for early and minimally invasive detection of RCC [1,9]. MiRNAs are small non-coding RNAs involved in cell differentiation, growth, apoptosis, and proliferation, and have been implicated in suppressing gene expression after translation [10,11]. MicroRNA dysregulation has been extensively described in various cancers, including RCC [4,10,11,12,13]. Frequently, miRNA levels differ between cancerous and normal tissues, representing an opportunity for biomarker development, both in tissue samples and in liquid biopsies [10,11]. Nonetheless, the biomarker performance of most candidate miRNAs remains suboptimal, and concerns remain as to the most adequate methods for assessment and normalization [14,15]. Indeed, all published studies on the assessment of miRNAs in the liquid biopsies of RCC patients have used qRT-PCR [1,9], a technique which provides relative quantification, thus requiring normalization of the results. Although miR-16 should be the preferential normalizer due to its stability in RCC [15,16,17,18], many of the published studies used RNU44, U6, or other similar RNA species instead, which are unstable in liquid biopsies, eventually leading to biased results [14,19,20,21,22,23,24,25,26,27]. This problem might be solved using a different technology, droplet digital PCR (ddPCR), as it obviates the need for normalization and preamplification. DdPCR is a recent technology that appears to improve miRNA detection, as it is based on sample partitioning before the PCR reaction and on the Poisson distribution, allowing for absolute quantification, in a time-cost effective and reliable manner [28,29]. Furthermore, the time point of data acquisition increases the precision and robustness of the method [28,29].

Thus, in this study, taking advantage of the performance of ddPCR in liquid biopsies, we sought to evaluate, for the first time, the ability of a microRNA panel (hsa-miR-21-5p, hsa-miR-126-3p, hsa-miR-155-5p, and hsa-miR-200b-3p), previously assessed in tissue samples [13,30] to detect RCC using plasma samples.

## 2. Materials and Methods

### 2.1. Samples

A total of five plasma samples were included in the technical optimization phase of the study, in which the ddPCR methodology was tested: one oncocytoma, one stage I pRCC, one stage I ccRCC, one stage I chRCC, and one healthy adult blood donor.

After optimizing the ddPCR pipeline, a cohort of 203 plasma samples was assessed, comprising 139 samples collected from RCT patients at the time of diagnosis and 64 healthy blood donors. Regarding RCT patients, 87 corresponded to ccRCC, 22 to chRCC and 15 to pRCC, whereas oncocytoma was diagnosed in the remaining 15. All patients were treated at IPO Porto by the same multidisciplinary team between 2015 and 2021. After peripheral blood collection into EDTA-containing tubes, plasma was separated by centrifugation at 2500 g for 30 min at 4 °C, and subsequently stored at −80 °C in the institutional biobank until further use. All blood samples were processed within 4 h from the time of collection. Relevant clinical and pathological data were analyzed from clinical charts and grouped in an anonymized database specifically constructed for the analysis.

### 2.2. RNA Extraction and cDNA Synthesis

Total RNA was extracted from 100 µL plasma using a MagMAX mirVana Total RNA Isolation kit (Thermo Fisher, Waltham, MA, USA, A27828), according to the manufacturer’s protocol. As a technical control, a non-human synthetic spike-in, ath-miR-159a (0.2 µL per sample of a stock solution at 0.2 nM), was added to the lysis buffer in all samples. The final 50 µL of RNA was collected to a 1.5 mL RNase-free tube. All steps were performed at room temperature, and extracted RNA was stored at −80 °C until further use.

Using TaqMan microRNA reverse transcription kit (Thermo Fisher, 4366596) according to the manufacturer’s protocol, five microliters of previously isolated RNA were reversely transcribed in a Veriti thermal cycler (Applied Biosystems^TM^, Waltham, MA, USA) for the miRNAs of interest and the spike-in (ath-miR-159a, hsa-miR-21-5p, hsa-miR-126-3p, hsa-miR-155-5p, hsa-miR-200b-3p).

### 2.3. Droplet Digital PCR (ddPCR): DigiMir Pipeline

DdPCR reactions were prepared according to the optimizations performed: the volumes of cDNA input [2 µL (ath-miR-159a, hsa-miR-21-5p, hsa-miR-126-3p), 5 µL (hsa-miR-155-5p and hsa-miR-200b-3p)], 11 µL ddPCR Supermix for the probes (Bio-Rad, Hercules, CA, USA, #1863010), and 1 µL TaqMan hsa-miRNA Assay (20×). The volumes of bidistilled water were 8 µL (ath-miR-159a, hsa-miR-21-5p, hsa-miR-126-3p) and 5 µL (hsa-miR-155-5p and hsa-miR-200b-3p); assays: ath-miR-159a—000338, FAM; hsa-miR-21-5p—000397, FAM; hsa-miR-126-3p—002228, VIC; hsa-miR-155-5p—002623, FAM; hsa-miR-200b-3p—002251, FAM. Droplets were generated on the droplet generator QX200 (Bio-Rad, Hercules, CA, USA). The PCR run was set as follows: 95 °C for 10 min, 50 cycles of 94 °C for 30 s, and “Annealing Temperature optimized” for 1 min—ramp rate 2 °C/s—and 98 °C for 10 min. The Annealing Temperature was set at 56 °C for ath-miR-159a and at 55 °C for the other four miRNAs. After PCR reaction, plate was read on the QX200 Droplet Reader (Bio-Rad, Hercules, CA, USA).

The limit of the blank (LOB) and the limit of detection (LOD) were calculated for each target miRNA according to Armsbruster et al. [2]. Additionally, the limit of quantification (LOQ) for the five miRNAs was assessed by performing a 2-fold dilution series of an RCT sample.

### 2.4. Quality Control Steps

All plasma samples were inspected for hemolysis as previously reported by others [31,32]. Hence, from 238 initial samples, 35 samples that presented absorbance higher than 0.25 at 414 nm were excluded. Appropriate engineering and manual controls were used to prevent contaminations—including a master mix made using a clean hood, clean gloves, PCR reagents and consumables—and reactions were performed in separate dedicated labs. RNA previously extracted from RCC cell lines (HKC8 was obtained from Expasy and Caki-1, 769-P, Caki-2, ACHN, A-498, HEK-293, 786-O were from ATCC), and a pool of them was used as positive control for the four candidate miRNAs. A no-template control (NTC) and no-enzyme control (NEC) were included in all cDNA synthesis and ddPCR stages as negative controls. For ddPCR pipeline optimization, further negative controls (“no-cDNA control” and “no-Supermix control”) were included, as recommended [33]. All samples were run in a single reaction for each target.

### 2.5. Statistical Analysis

Non-parametric tests were performed to compare levels of each miRNA among histologic subtypes and to evaluate associations with clinicopathological features. A Spearman test was used for correlation analyses between two variables. A Mann-Whitney U test was used for comparisons between two groups, whereas a Kruskal-Wallis test was used for multiple groups, followed by a Mann-Whitney U test with Bonferroni’s correction for pairwise comparisons. A result was considered statistically significant when the *p*-value < 0.05.

For each miRNA, samples were categorized as positive or negative based on the cut-off values established using Youden’s J index [34,35] (value combining the highest sensitivity and specificity), through Receiver-Operating Characteristic (ROC) curve analysis. Validity estimates (sensitivity, specificity, and accuracy) were determined to assess the detection biomarker performance. To improve the detection performance of the selected miRNAs, panels were constructed considering a positive result whenever at least one target miRNA was plotted as positive in an individual analysis.

A two-tailed *p*-value calculation and ROC curve analyses (without resampling analysis) were performed using SPSS 27.0 software for Windows (IBM-SPSS Inc., Chicago, IL, USA). All graphics were assembled using GraphPad Prism 8.0 software for Windows (GraphPad Software Inc., LA Jolla, CA, USA). To increase the statistical power through a resampling analysis, multiple ROC curves were constructed to calculate validity estimates for the best miRNA panels, as previously described [36,37]. In brief, samples were randomly divided into training (70%) and validation (30%) sets. Subsequently, the cut-off value was estimated in the training set considering the highest sensitivity and specificity and using this calculated cut-off, validity estimates were calculated in the validation set. The procedure was repeated 1000 times and the mean of the parameters (sensitivity and specificity) were calculated. These calculations were performed using R v3.4.4.

## 3. Results

### 3.1. Patients’ Cohort Characterization

The relevant clinical-pathological features of optimization and validation cohorts are depicted in Table 1.

According to clinical-demographic factors, a significant, although weak, correlation was found between age and circulating levels of each miRNA—hsa-miR-21-5p, hsa-miR-126-3p and hsa-miR-200b-3p levels (R^2^ = 0.080 and *p*-value < 0.001, R^2^ = 0.030 and *p*-value = 0.023, R^2^ = 0.020 and *p*-value = 0.032, respectively).

### 3.2. Distribution of Circulating miRNA Levels and Biomarkers Performance for Detection of Malignancy

Initially, target miRNA levels were compared between oncocytoma (a benign tumor) and healthy donor samples, and no significant differences between these groups were found for any of the tested hsa-miRNAs, except for hsa-miR-155-5p (*p*-value = 0.037).

**Figure 1:**
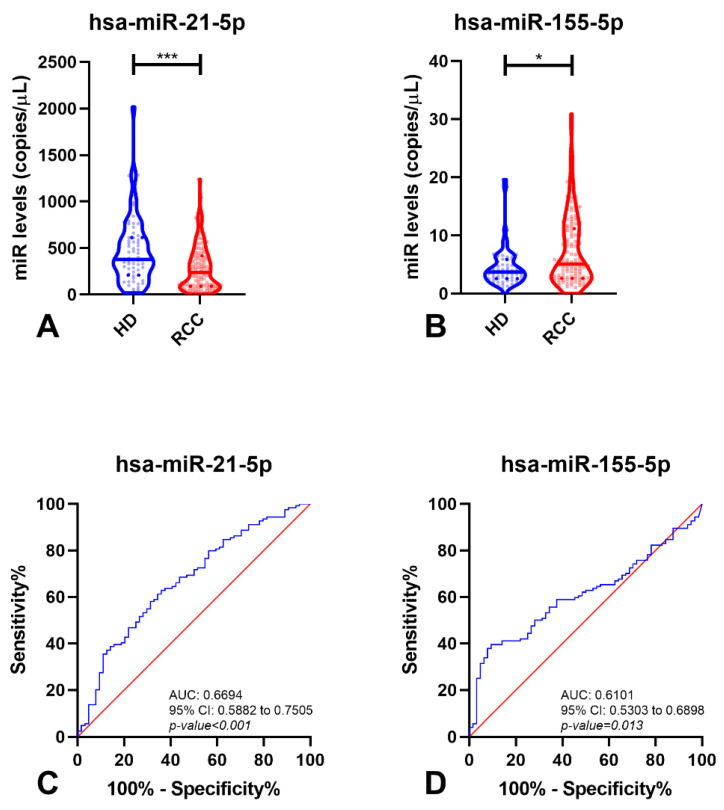
Violin plots with miRNA levels in Healthy Donors (HD) and Renal Cell Carcinoma (RCC) samples of hsa-miR-21-5p (**A**) and hsa-miR-155-5p (**B**), and respective Receiver-Operating Characteristic Curve (without resampling analysis) (**C**,**D**). In violin plots, dashed lines indicate the interquartile range and horizontal line the median of miR levels. In ROC curves, red line indicates the reference line and blue line the identity line for each miRNA. Abbreviations: AUC—Area Under the Curve; CI—Confidence Interval, HD—Healthy Donors, RCC—Renal Cell Carcinoma, *—*p*-value < 0.05, ***—*p*-value < 0.0001.

Due to the clinical relevance of discriminating malignant disease (RCC) from healthy individuals, this comparison was subsequently performed. Interestingly, circulating levels of hsa-miR-21-5p and hsa-miR-155-5p significantly differed between these two groups (*p*-value < 0.001 and *p*-value = 0.013, respectively) (Figure 1). Circulating levels of hsa-miR-21-5p disclosed the highest accuracy for identifying malignant tumors, although hsa-miR-155-5p depicted the best specificity (90.63%). Remarkably, a panel comprising hsa-miR-21-5p/hsa-miR-155-5p detected about 83% of the three major RCC subtypes, with 71.89% accuracy (Table 2). Importantly, the same two hsa-miRNAs could discriminate RCTs from healthy individuals (Figures S1 and S2 and Table S1).

**Figure 2:**
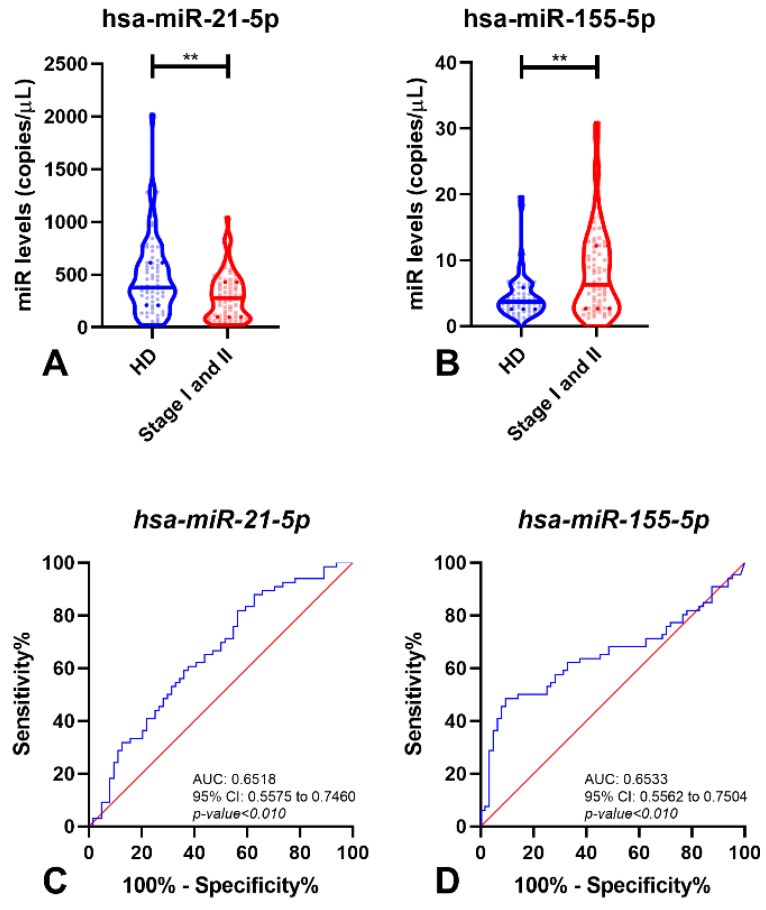
Violin plots of miRNAs levels in Healthy Donor (HD) and early stages of Renal Cell Carcinoma (Stage I and II) samples of hsa-miR-21-5p (**A**) and hsa-miR-155-5p (**B**), and respective Receiver-Operating Characteristic Curve (without resampling analysis) (**C**,**D**). In violin plots, dashed lines indicate the interquartile range and horizontal line the median of miR levels. In ROC curves, red line indicates the reference line and blue line the identity line for each miRNA. Abbreviations: AUC—Area Under the Curve; CI—Confidence Interval; HD—Healthy Donors, **—*p*-value < 0.001.

When the analysis was restricted to early-stage disease (patients with an organ-confined tumor) and healthy donor samples, hsa-miR-21-5p and hsa-miR-155-5p, but not the other miRNAs, retained statistical difference (*p*-value < 0.01 for both miRNAs) between these two groups (Figure 2A,B). Hence, these two miRNAs were able to detect small RCC (tumors limited to the kidney, without regional lymph node metastasis) with 89.04% sensitivity and high negative predictive value (NPV) (77.68%) (Table 3). Remarkably, the AUC for both miRNAs was higher than 65.00% (Figure 2C,D).

### 3.3. MiRNA Levels and Clinicopathological Features

**Figure 3:**
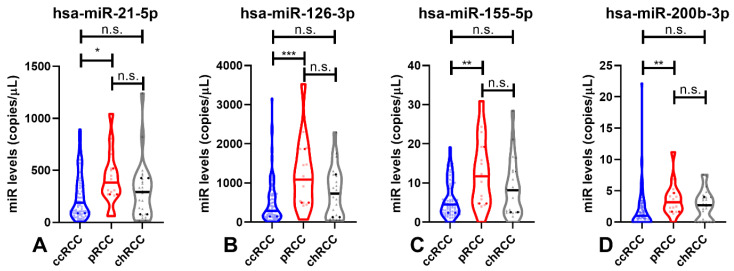
Violin plots of hsa-miR-21-5p (**A**), hsa-miR-126-3p (**B**), hsa-miR-155-5p (**C**) and hsa-miR-200b-3p (**D**) levels in the malignant subtypes (ccRCC, pRCC and chRCC). Dashed lines indicate the interquartile range and horizontal line the median of miR levels. Abbreviations: ccRCC—Clear-Cell Renal Cell Carcinoma; chRCC—Chromophobe Renal Cell Carcinoma; pRCC—Papillary Renal Cell Carcinoma; n.s.—not significant, *—*p*-value < 0.05, **—*p*-value < 0.001, ***—*p*-value < 0.0001.

Among RCC subtypes (ccRCC, pRCC and chRCC), significant differences were found for all four miRNAs (hsa-miR-126-3p, hsa-miR-155-5p and hsa-miR-200b-3p, *p*-value < 0.010; hsa-miR-21-3p, *p*-value = 0.045, Figure 3).

Furthermore, all four hsa-miRs circulating levels significantly differed between the two major RCC subtypes, ccRCC and pRCC (hsa-miR-126-3p, *p*-value < 0.001; hsa-miR-155-5p and hsa-miR-200b-3p, *p*-value < 0.01; hsa-miR-21-5p, *p*-value = 0.039, Figure 3). Nonetheless, no statistical differences were found between pRCC and chRCC or between ccRCC and chRCC for the tested circulating miRNAs.

**Figure 4:**
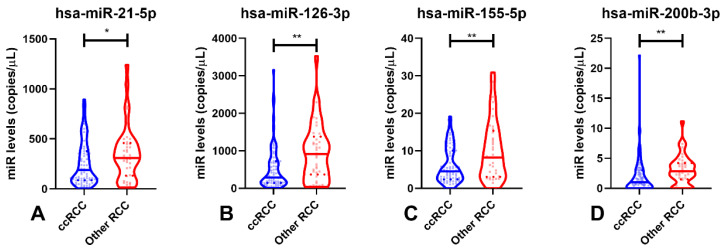
Violin plots of hsa-miR-21-5p (**A**), hsa-miR-126-3p (**B**), hsa-miR-155-5p (**C**) and hsa-miR-200b-3p (**D**) levels in ccRCC and other RCCs (pRCC and chRCC). Dashed lines indicate the interquartile range and horizontal line the median of miR levels. Abbreviations: ccRCC—Clear-Cell Renal Cell Carcinoma; RCC—Renal Cell Carcinomas; n.s.—not significant, *—*p*-value < 0.05, **—*p*-value < 0.001.

Due to the poorer outcome and higher incidence of ccRCC, comparisons in circulating hsa-miRNAs were performed between this subtype and the other two RCC subtypes (Figure 4). Interestingly, ccRCC patients displayed significantly lower circulating levels of all hsa-miRs compared to patients diagnosed with the other malignant subtypes (*p*-value = 0.048 for hsa-miR-21-5p and *p*-value < 0.01 for hsa-miR-126-3p, hsa-miR-155-5p and hsa-miR-200b-3p—Figure 4).

**Figure 5:**
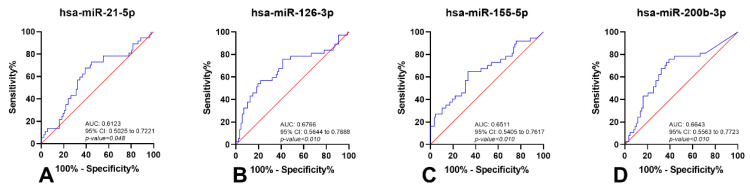
Receiver-Operating Characteristic Curves (without resampling analysis) of hsa-miR-21-5p (**A**), hsa-miR-126-3p (**B**), hsa-miR-155-5p (**C**) and hsa-miR-200b-3p (**D**) in ccRCC and other RCCs (pRCC and chRCC). Red line indicates the reference line and blue line the identity line for each miRNA. Abbreviations: AUC—Area Under the Curve; CI—Confidence Interval.

Moreover, circulating hsa-miR-126-3p and hsa-miR-200b-3p levels discriminated ccRCC from other RCC subtypes with 74.78% sensitivity and 52.95% specificity (Figure 5 and Table 4).

## 4. Discussion

RCC remains a leading cause of cancer-related death worldwide. Alongside prostate and bladder cancers, RCC is one of the most common urological malignancies [38]. Early detection of RCC (ideally at stage I or II) significantly increases the likelihood of a cure through surgical treatment, with a 5-year survival rate of 98%, averting the need for subsequent therapies, which are not curative and often carry significant adverse side effects [15]. Nonetheless, 20–30% of patients display metastatic disease at diagnosis [38,39], and even following curative-intent nephrectomy, the standard of care for localized RCC, metastases develop in up to 20–40% of patients [39]. Notably, the response to medical treatment (mainly targeted therapy or immunotherapy) is rather limited, with a 5-year survival rate lower than 10%. Among RCCs, ccRCC, pRCC, and chRCC represent more than 90% of cases, emphasizing the importance of accurately detecting these tumor subtypes and discriminating them from benign conditions [39,40].

Circulating miRNAs are emergent cancer biomarkers which might be assessed using minimally invasive strategies, eventually constituting promising RCC biomarkers. Nevertheless, only a few studies have addressed this issue, mostly using conventional qPCR techniques [14,15,17,18,19,20,21,22,23,24,25,41,42]. Owing to the diversity of the results of those studies and the need to overcome the limitations of normalization, we assessed the clinical potential of a circulating miRNA-based panel for RCC detection using ddPCR.

Accurate identification of patients harboring RCC and discrimination from healthy individuals, as well as from carriers of benign renal lesions (including tumors), is pivotal to reliably establishing therapeutic vs. monitoring strategies. Thus, after a first analysis between oncocytomas and healthy donors, we compared healthy donors with RCC patients. Remarkably, two (hsa-miR-21-5p and hsa-miR-155-5p) out of the four candidate miRNAs disclosed statistically significant differences in plasma levels. Although hsa-miR-21-5p has been described to act as oncomiR, we observed lower circulating levels in RCC patients [20,43,44,45]. This might be due to the distinct miRNAs levels in the different clinical samples. Indeed, higher miRNA levels may be found in tissues compared to body fluid samples [46]. Importantly, increased hsa-miR-21-5p levels were also found in serum samples of RCC patients, further supporting that circulating miRNA levels in serum and plasma may be different [20]. Moreover, differences were also reported for hsa-miR-21-5p levels in serum and plasma among patients with Non-ST-elevation myocardial infarction, a non-cancer-related pathology [47]. Herein, higher hsa-miR-21-5p levels were found in serum when compared with respective control samples, whereas lower levels were observed in plasma samples from the same patients [47]. Of note, plasma has been reported to be the sample of election for translational studies [47,48,49], as red blood cell lysis during the coagulation process increases discharging of RNA and platelets to the serum, increasing the non-tumor derived circulating miRNAs present in each sample [48]. Importantly, hsa-miR-21-5p is expressed in platelets [47,50] and, thus, an increase of platelets in serum might explain the higher levels found for this miRNA. Furthermore, in breast cancer, lower hsa-miR-30b-5p levels were found in tissue compared with plasma, unveiling the disparities between these two sample sources [51]. Moreover, inadequate normalization and biased results may occur if the normalizer used is not the most suitable. Indeed, U6 is more prone to degradation by serum RNases [1]. Interestingly, in a previous study we found that hsa-miR-21-5p miRNA was significantly downregulated in tissue samples from RCT patients, discriminating RCT patients from healthy donors [13].

Concerning hsa-miR-155-5p, upregulation of this circulating hsa-miR was found in RCC patients, and a panel comprising hsa-miR-155-5p and hsa-miR-21-5p could identify 82.66% of RCC patients with 71.89% accuracy. Interestingly, hsa-miR-155-5p was shown to be upregulated in tissue [13,52] and ccRCC serum samples [18], and is also associated with cancer development [52]. Moreover, an hsa-miR-21-5p/hsa-miR-155-5p panel depicted high sensitivity (89.04%) for identifying organ-confined carcinomas, which might allow for reducing false-negative results and increase the likelihood of curative-intent treatment. To the best of our knowledge, this is the first study that evaluated the biomarker performance of plasma circulating hsa-miRs to detect early-stage RCC. Previously, Wang and colleagues described a 5-miRNA panel (miR-193a-3p, miR-362, miR-572, miR-378, and miR-28-5p) that was able to identify early-stage RCC, albeit in serum samples [22]. Furthermore, our panel achieved a higher NPV than that reported by Wang et al. [22].

We further evaluated whether circulating hsa-miRNAs might also convey relevant information to discriminate ccRCC from the remainder RCC subtypes. Indeed, all four miRNAs were able to differentiate this major RCC subtype from the others. The panel constituted by hsa-miR-126-3p and hsa-miR-200b-3p disclosed the best performance, with 74.78% sensitivity and 52.95% specificity. Since ccRCC is an aggressive RCC subtype, early detection is of major importance, and its accurate identification might improve patient outcomes [20,53]. Although stratification by stage was not performed due to a limited number of cases with advanced stages, for early stages, hsa-miR-126-3p and hsa-miR-200b-3p levels also differed significantly between ccRCC and the remainder RCC subtypes.

Considering that various studies have reported other strategies for RCC identification (including imaging and epigenetic biomarkers), our results seem to offer the best sensitivity for RCC detection [9,54]. Indeed, the methodology we developed uses a lower initial sample volume [15,17,20,22,25,41,42], which is more cost-effective, and the procedure to obtain the sample is better tolerated by patients. Molecular imaging such as ^18^F-fluorodeoxt-glucose (FDG) positron emission tomography/computed tomography (PET/CT) was reported to detect localized RCC, but it discloses lower sensitivity (only 22%) [54,55]. Despite the superior specificity (85.9%) of ^124^I-cG250 PET for RCC detection, when compared to our hsa-miR-21-5p/hsa-miR-155-5p panel (51.13%), this monoclonal antibody has a half-life of several days, constituting a significant disadvantage in relation to the protocol reported by us [56]. Moreover, diffusion magnetic resonance imaging was reported to characterize malignant lesions with similar sensitivity (86%) to our panel but with higher specificity (78%) [57]. Nevertheless, it should be noted that despite the better performance, these imaging biomarkers are more costly and less well-tolerated by the patient compared to liquid biopsies [54].

The intense exploration of circulating epigenetic markers such as DNA methylation, miRNAs, and lncRNAs is well illustrated by the more that 60 articles published in this field since 2003 [9]. So far, 10 DNA methylation-based studies (e.g., using *VHL, RASSF1A, P16, P14, RARB, TIMP3, GSTP1, APC*) for RCC detection have been published [58,59,60,61,62,63,64,65,66,67] and only 33.33% of these had an RCC cohort with more than 50 patients [60,63,64]. Compared with those studies, our results provide higher sensitivity (6–83%). However, DNA methylation-based markers displayed high specificity (53–100%). This was also observed in three lncRNAs studies (e.g., GIHCG, LINC00887) [68,69,70], in which the diagnostic performance was generally lower than in our study (67.1–87.0%), but the specificity reached values >80% for all biomarkers. Although our biomarker panels disclosed high sensitivity, their specificity is limited. Thus, in an envisaged routine setting, they would ideally be used in first-line screening, requiring complementary use of more specific biomarkers in cases deemed positive. In liquid biopsies, DNA methylation-based markers such as VHL, RASSF1A, TIMP3, SFRP1, SFRP2, SFRP4, SFRP5, PCDH17, and TCF21 are highly specific (100%) [58,59,61,62,65,66,67] and, thus, constitute good candidates as second-line tests, in this setting.

**Figure 6:**
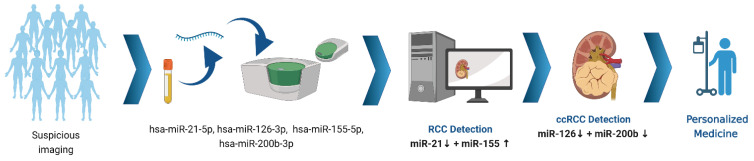
Graphical representation of the potential clinical impact of LiKidMiRs. Created with BioRender.com.

As previously reported, most circulating miRNA studies are based on blood-based liquid biopsies [1]. When compared with our protocol, only a few studies included more than 100 RCC patients, which might, at the least partially, explain the differences in results [9]. Additionally, the discrepant results might also be explained, as described above, by the biased normalization (e.g., spike-in as normalizer miRNA, U6, RNU48) [14,19,20,23,24]. Nevertheless, the sensitivity reported for the most widely studied serum miRNAs (miR-210, miR-1233, and miR-378) was generally lower than our plasma panel [14,17,25]. Indeed, using this less time-consuming and more cost-effective approach, we were able to detect RCC using a minimally invasive technique, with a lower initial quantity of plasma than serum-based studies (although detecting other miRNAs), and obtained similar or even better results, obviating the need for normalization and the associated bias (due to ddPCR absolute quantification) [15,17,20,22,25,41,42]. Hence, our results from multiple ROC curve analysis demonstrate a potential clinical application of this technology to identify RCC, and is the first study to quantify circulating miRNAs in these patients using ddPCR (Figure 6).

These results require validation in more extensive prospective studies. Overall, and notwithstanding our promising results for RCC detection, it should be acknowledged that the lack of long-term follow-up constitutes a significant limitation. Further studies using liquid biopsies should also be considered to further subtype RCC, namely, to distinguish oncocytomas from chRCCs, which will lead to a prioritization of treatments for patients with malignant tumors.

## 5. Conclusions

Our findings support the research question that a minimally invasive test can be developed to detect RCC, improving patient survival through increased diagnosis at earlier stages. This might help to reduce the morbidity and mortality associated with advanced disease, as well as the lack of curative treatment at those stages. Furthermore, and to the best of our knowledge, this work is the first to report a novel tool to quantify circulating miRNAs in plasma using ddPCR in RCC patients.

## Figures and Tables

**Table 1:** Clinicopathological data of the technical optimization cohort (5 samples) and LiKidMiRs cohort (composed of 139 Renal Cell Tumors and 64 Healthy donors’ samples) used in this study.

Technical Optimization Cohort (*n* = 5 Samples)	
Cases	Description
Sample #1	66 years, Oncocytoma
Sample #2	53 years, pRCC, Stage I
Sample #3	57 years, ccRCC, Stage I
Sample #4	46 years, chRCC, stage I
Sample #5	45 years, healthy blood donor
**LiKidMiRs Cohort (*n* = 203 samples)**	
Renal cell tumor samples	139
Healthy blood donors	64
Renal cell tumor patients—clinicopathological features	
Age [years (median, interquartile range)]	64 (17.0)
Gender	
Male	96/139 (69.1)
Female	43/139 (30.9)
Size of tumor mass [cm (median, interquartile range)]	4.50 (4.3)
Histology [*n*, (%)]	
ccRCC	87/139 (62.6)
pRCC	15/139 (10.8)
chRCC	22/139 (15.8)
Oncocytoma	15/139 (10.8)
Stage [*n*, (%)]	
I	59/124 (47.6)
II	8/124 (6.5)
III	45/124 (36.3)
IV	12/124 (9.7)
ISUP nuclear grade [*n*, (%)]	
1	7/88 (8.0)
2	47/88 (53.4)
3	24/88 (27.3)
4	10/88 (11.4)
Vital status	
Alive with disease	6/139 (4.3)
Alive without disease	120/139 (86.3)
Death from the disease	13/139 (9.4)
Healthy Blood Donors—clinicopathological features	
Age [years (median, interquartile range)]	46 (4.75)
Gender	
Male	36/64 (56.3)
Female	28/64 (43.8)

**Table 2:** Performance of miRNAs as biomarkers for detection of Renal Cell Carcinoma.

miRNAs	SE%	SP%	PPV%	NPV%	Accuracy%
hsa-miR-21-5p	62.90	64.06	77.23	47.13	63.30
hsa-miR-155-5p	39.52	90.63	89.09	43.61	56.91
hsa-miR-21-5p/hsa-miR-155-5p	89.52	54.69	79.29	72.92	77.66
Multiple ROC Curve (hsa-miR-21-5p/hsa-miR-155-5p)	82.66	51.13	77.22	61.76	71.89

Abbreviations: SE—Sensitivity; SP—Specificity; PPV—Positive Predictive Value; NPV—Negative Predictive Value; ROC—Receiver-Operating Characteristic.

**Table 3:** Performance of miRNAs as biomarkers for identification of early stages Renal Cell Carcinomas.

miRNAs	SE%	SP%	PPV%	NPV%	Accuracy%
hsa-miR-21-5p	81.82	43.75	60.00	70.00	63.08
hsa-miR-155-5p	48.48	90.63	84.21	63.04	69.23
hsa-miR-21-5p/hsa-miR-155-5p	92.42	34.38	59.22	81.48	63.85
Multiple ROC Curve (hsa-miR-21-5p/hsa-miR-155-5p)	89.04	36.23	59.28	77.68	62.88

Abbreviations: SE—Sensitivity; SP—Specificity; PPV—Positive Predictive Value; NPV—Negative Predictive Value; ROC—Receiver-Operating Characteristic.

**Table 4:** Performance of miRNAs as biomarkers for identification of Clear-Cell Renal Cell Carcinoma.

miRNAs	SE%	SP%	PPV%	NPV%	Accuracy%
hsa-miR-21-5p	60.92	67.57	81.54	42.37	62.90
hsa-miR-126-3p	78.16	56.76	80.95	52.50	71.77
hsa-miR-155-5p	66.67	64.86	81.69	45.28	66.13
hsa-miR-200b-3p	60.92	75.68	85.48	45.16	65.32
hsa-miR-126-3p/hsa-miR-200b-3p	80.46	56.76	81.40	55.26	73.39
Multiple ROC Curve (hsa-miR-126-3p/hsa-miR-200b-3p)	74.78	52.95	79.49	47.46	68.28

Abbreviations: SE—Sensitivity; SP—Specificity; PPV—Positive Predictive Value; NPV—Negative Predictive Value.

## Data Availability

All data generated or analyzed during this study are included in this article.

## Supplementary Materials

The following are available online at https://www.mdpi.com/article/10.3390/cancers14040858/s1, Table S1: Performance of miRNAs as biomarkers for detection of Renal Cell Tumors., Figure S1: Violin plots with all points of miRNAs levels in Healthy Donors (HD) and Renal Cell Tumors (RCT) samples of hsa-miR-21-5p (A), hsa-miR-126-3p (B), hsa-miR-155-5p (C) and hsa-miR-200b-3p (D). Dashed lines indicate the interquartile range and horizontal line the median of miR levels. Abbreviations: HD—Healthy Donors; RCT—Renal Cell Tumors; n.s.—not significant, **—*p*-value < 0.001, ***—*p*-value < 0.0001., Figure S2: Receiver Operating Characteristic Curve (without resampling analysis) of hsa-miR-21-5p (A) and hsa-miR-155-5p (B). Red line indicates the reference line and blue line the identity line for each miRNA. Abbreviations: AUC—Area Under the Curve; CI—Confidence Interval.

## References

[1] Sequeira J.P., Constâncio V., Lobo J., Henrique R., Jerónimo C. (2021). Unveiling the World of Circulating and Exosomal microRNAs in Renal Cell Carcinoma. Cancers.

[2] Sung H., Ferlay J., Siegel R.L., Laversanne M., Soerjomataram I., Jemal A., Bray F. (2021). Global Cancer Statistics 2020: GLOBOCAN Estimates of Incidence and Mortality Worldwide for 36 Cancers in 185 Countries. CA Cancer J. Clin..

[3] Maher E.R. (2013). Genomics and epigenomics of renal cell carcinoma. Semin. Cancer Biol..

[4] Outeiro-Pinho G., Barros-Silva D., Correia M.P., Henrique R., Jerónimo C. (2020). Renal Cell Tumors: Uncovering the Biomarker Potential of ncRNAs. Cancers.

[5] Shuch B., Amin A., Armstrong A.J., Eble J.N., Ficarra V., Lopez-Beltran A., Martignoni G., Rini B.I., Kutikov A. (2015). Understanding pathologic variants of renal cell carcinoma: Distilling therapeutic opportunities from biologic complexity. Eur. Urol..

[6] Arora R.D., Limaiem F. (2021). Renal Clear Cell Cancer. StatPearls.

[7] Pandey J., Syed W. (2021). Renal Cancer. StatPearls.

[8] Ricketts C.J., De Cubas A.A., Fan H., Smith C.C., Lang M., Reznik E., Bowlby R., Gibb E.A., Akbani R., Beroukhim R. (2018). The Cancer Genome Atlas Comprehensive Molecular Characterization of Renal Cell Carcinoma. Cell Rep..

[9] Kubiliute R., Jarmalaite S. (2021). Epigenetic Biomarkers of Renal Cell Carcinoma for Liquid Biopsy Tests. Int. J. Mol. Sci..

[10] Filella X., Foj L. (2017). miRNAs as novel biomarkers in the management of prostate cancer. Clin. Chem. Lab. Med..

[11] Lu J., Getz G., Miska E.A., Alvarez-Saavedra E., Lamb J., Peck D., Sweet-Cordero A., Ebert B.L., Mak R.H., Ferrando A.A. (2005). MicroRNA expression profiles classify human cancers. Nature.

[12] Guil S., Esteller M. (2009). DNA methylomes, histone codes and miRNAs: Tying it all together. Int. J. Biochem. Cell Biol..

[13] Silva-Santos R.M., Costa-Pinheiro P., Luis A., Antunes L., Lobo F., Oliveira J., Henrique R., Jeronimo C. (2013). MicroRNA profile: A promising ancillary tool for accurate renal cell tumour diagnosis. Br. J. Cancer.

[14] Wulfken L.M., Moritz R., Ohlmann C., Holdenrieder S., Jung V., Becker F., Herrmann E., Walgenbach-Brünagel G., von Ruecker A., Müller S.C. (2011). MicroRNAs in Renal Cell Carcinoma: Diagnostic Implications of Serum miR-1233 Levels. PLoS ONE.

[15] Iwamoto H., Kanda Y., Sejima T., Osaki M., Okada F., Takenaka A. (2014). Serum miR-210 as a potential biomarker of early clear cell renal cell carcinoma. Int. J. Oncol..

[16] Heinemann F.G., Tolkach Y., Deng M., Schmidt D., Perner S., Kristiansen G., Müller S.C., Ellinger J. (2018). Serum miR-122-5p and miR-206 expression: Non-invasive prognostic biomarkers for renal cell carcinoma. Clin. Epigenetics.

[17] Redova M., Poprach A., Nekvindova J., Iliev R., Radova L., Lakomy R., Svoboda M., Vyzula R., Slaby O. (2012). Circulating miR-378 and miR-451 in serum are potential biomarkers for renal cell carcinoma. J. Transl. Med..

[18] Wang X., Wang T., Chen C., Wu Z., Bai P., Li S., Chen B., Liu R., Zhang K., Li W. (2018). Serum exosomal miR-210 as a potential biomarker for clear cell renal cell carcinoma. J. Cell Biochem..

[19] Mytsyk Y., Dosenko V., Borys Y., Kucher A., Gazdikova K., Busselberg D., Caprnda M., Kruzliak P., Farooqi A.A., Lubov M. (2018). MicroRNA-15a expression measured in urine samples as a potential biomarker of renal cell carcinoma. Int. Urol. Nephrol..

[20] Tusong H., Maolakuerban N., Guan J., Rexiati M., Wang W.G., Azhati B., Nuerrula Y., Wang Y.J. (2017). Functional analysis of serum microRNAs miR-21 and miR-106a in renal cell carcinoma. Cancer Biomark..

[21] von Brandenstein M., Pandarakalam J.J., Kroon L., Loeser H., Herden J., Braun G., Wendland K., Dienes H.P., Engelmann U., Fries J.W. (2012). MicroRNA 15a, inversely correlated to PKCα, is a potential marker to differentiate between benign and malignant renal tumors in biopsy and urine samples. Am. J. Pathol..

[22] Wang C., Hu J., Lu M., Gu H., Zhou X., Chen X., Zen K., Zhang C.-Y., Zhang T., Ge J. (2015). A panel of five serum miRNAs as a potential diagnostic tool for early-stage renal cell carcinoma. Sci. Rep..

[23] Yadav S., Khandelwal M., Seth A., Saini A.K., Dogra P.N., Sharma A. (2017). Serum microRNA Expression Profiling: Potential Diagnostic Implications of a Panel of Serum microRNAs for Clear Cell Renal Cell Cancer. Urology.

[24] Zhai Q., Zhou L., Zhao C., Wan J., Yu Z., Guo X., Qin J., Chen J., Lu R. (2012). Identification of miR-508-3p and miR-509-3p that are associated with cell invasion and migration and involved in the apoptosis of renal cell carcinoma. Biochem. Biophys. Res. Commun..

[25] Zhao A., Li G., Péoc’h M., Genin C., Gigante M. (2013). Serum miR-210 as a novel biomarker for molecular diagnosis of clear cell renal cell carcinoma. Exp. Mol. Pathol..

[26] Teixeira A.L., Ferreira M., Silva J., Gomes M., Dias F., Santos J.I., Maurício J., Lobo F., Medeiros R. (2014). Higher circulating expression levels of miR-221 associated with poor overall survival in renal cell carcinoma patients. Tumour Biol..

[27] Zhang Q., Di W., Dong Y., Lu G., Yu J., Li J., Li P. (2015). High serum miR-183 level is associated with poor responsiveness of renal cancer to natural killer cells. Tumour Biol..

[28] Campomenosi P., Gini E., Noonan D.M., Poli A., D’Antona P., Rotolo N., Dominioni L., Imperatori A. (2016). A comparison between quantitative PCR and droplet digital PCR technologies for circulating microRNA quantification in human lung cancer. BMC Biotechnol..

[29] Taylor S.C., Laperriere G., Germain H. (2017). Droplet Digital PCR versus qPCR for gene expression analysis with low abundant targets: From variable nonsense to publication quality data. Sci. Rep..

[30] Di Meo A., Saleeb R., Wala S.J., Khella H.W., Ding Q., Zhai H., Krishan K., Krizova A., Gabril M., Evans A. (2017). A miRNA-based classification of renal cell carcinoma subtypes by PCR and in situ hybridization. Oncotarget.

[31] Androvic P., Romanyuk N., Urdzikova-Machova L., Rohlova E., Kubista M., Valihrach L. (2019). Two-tailed RT-qPCR panel for quality control of circulating microRNA studies. Sci. Rep..

[32] van Vliet E.A., Puhakka N., Mills J.D., Srivastava P.K., Johnson M.R., Roncon P., Das Gupta S., Karttunen J., Simonato M., Lukasiuk K. (2017). Standardization procedure for plasma biomarker analysis in rat models of epileptogenesis: Focus on circulating microRNAs. Epilepsia.

[33] Stein E.V., Duewer D.L., Farkas N., Romsos E.L., Wang L., Cole K.D. (2017). Steps to achieve quantitative measurements of microRNA using two step droplet digital PCR. PLoS ONE.

[34] Schisterman E.F., Perkins N.J., Liu A., Bondell H. (2005). Optimal cut-point and its corresponding Youden Index to discriminate individuals using pooled blood samples. Epidemiology.

[35] Youden W.J. (1950). Index for rating diagnostic tests. Cancer.

[36] Baker S.G., Kramer B.S. (2006). Identifying genes that contribute most to good classification in microarrays. BMC Bioinform..

[37] Nunes S.P., Moreira-Barbosa C., Salta S., Palma de Sousa S., Pousa I., Oliveira J., Soares M., Rego L., Dias T., Rodrigues J. (2018). Cell-Free DNA Methylation of Selected Genes Allows for Early Detection of the Major Cancers in Women. Cancers.

[38] Fan B., Jin Y., Zhang H., Zhao R., Sun M., Sun M., Yuan X., Wang W., Wang X., Chen Z. (2020). MicroRNA-21 contributes to renal cell carcinoma cell invasiveness and angiogenesis via the PDCD4/c-Jun (AP-1) signalling pathway. Int. J. Oncol..

[39] Carlsson J., Christiansen J., Davidsson S., Giunchi F., Fiorentino M., Sundqvist P. (2019). The potential role of miR-126, miR-21 and miR-10b as prognostic biomarkers in renal cell carcinoma. Oncol. Lett.

[40] Lopez-Beltran A., Carrasco J.C., Cheng L., Scarpelli M., Kirkali Z., Montironi R. (2009). 2009 update on the classification of renal epithelial tumors in adults. Int. J. Urol..

[41] Fedorko M., Juracek J., Stanik M., Svoboda M., Poprach A., Buchler T., Pacik D., Dolezel J., Slaby O. (2017). Detection of let-7 miRNAs in urine supernatant as potential diagnostic approach in non-metastatic clear-cell renal cell carcinoma. Biochem. Med..

[42] Fedorko M., Stanik M., Iliev R., Redova-Lojova M., Machackova T., Svoboda M., Pacik D., Dolezel J., Slaby O. (2015). Combination of MiR-378 and MiR-210 Serum Levels Enables Sensitive Detection of Renal Cell Carcinoma. Int. J. Mol. Sci..

[43] Chen J., Gu Y., Shen W. (2017). MicroRNA-21 functions as an oncogene and promotes cell proliferation and invasion via TIMP3 in renal cancer. Eur. Rev. Med. Pharmacol. Sci..

[44] Jung M., Mollenkopf H.J., Grimm C., Wagner I., Albrecht M., Waller T., Pilarsky C., Johannsen M., Stephan C., Lehrach H. (2009). MicroRNA profiling of clear cell renal cell cancer identifies a robust signature to define renal malignancy. J. Cell. Mol. Med..

[45] Lokeshwar S.D., Talukder A., Yates T.J., Hennig M.J.P., Garcia-Roig M., Lahorewala S.S., Mullani N.N., Klaassen Z., Kava B.R., Manoharan M. (2018). Molecular Characterization of Renal Cell Carcinoma: A Potential Three-MicroRNA Prognostic Signature. Cancer Epidemiol. Biomark. Prev..

[46] Nagy Z.B., Barták B.K., Kalmár A., Galamb O., Wichmann B., Dank M., Igaz P., Tulassay Z., Molnár B. (2019). Comparison of Circulating miRNAs Expression Alterations in Matched Tissue and Plasma Samples During Colorectal Cancer Progression. Pathol. Oncol. Res..

[47] Mompeón A., Ortega-Paz L., Vidal-Gómez X., Costa T.J., Pérez-Cremades D., Garcia-Blas S., Brugaletta S., Sanchis J., Sabate M., Novella S. (2020). Disparate miRNA expression in serum and plasma of patients with acute myocardial infarction: A systematic and paired comparative analysis. Sci. Rep..

[48] Wang K., Yuan Y., Cho J.-H., McClarty S., Baxter D., Galas D.J. (2012). Comparing the MicroRNA spectrum between serum and plasma. PLoS ONE.

[49] Dufourd T., Robil N., Mallet D., Carcenac C., Boulet S., Brishoual S., Rabois E., Houeto J.-L., de la Grange P., Carnicella S. (2019). Plasma or serum? A qualitative study on rodents and humans using high-throughput microRNA sequencing for circulating biomarkers. Biol. Methods Protoc..

[50] Willeit P., Zampetaki A., Dudek K., Kaudewitz D., King A., Kirkby N.S., Crosby-Nwaobi R., Prokopi M., Drozdov I., Langley S.R. (2013). Circulating MicroRNAs as Novel Biomarkers for Platelet Activation. Circ. Res..

[51] Adam-Artigues A., Garrido-Cano I., Simón S., Ortega B., Moragón S., Lameirinhas A., Constâncio V., Salta S., Burgués O., Bermejo B. (2021). Circulating miR-30b-5p levels in plasma as a novel potential biomarker for early detection of breast cancer. ESMO Open.

[52] Ji H., Tian D., Zhang B., Zhang Y., Yan D., Wu S. (2017). Overexpression of miR-155 in clear-cell renal cell carcinoma and its oncogenic effect through targeting FOXO3a. Exp. Ther. Med..

[53] Cheng T., Wang L., Li Y., Huang C., Zeng L., Yang J. (2013). Differential microRNA expression in renal cell carcinoma. Oncol. Lett..

[54] Farber N.J., Kim C.J., Modi P.K., Hon J.D., Sadimin E.T., Singer E.A. (2017). Renal cell carcinoma: The search for a reliable biomarker. Transl. Cancer Res..

[55] Gofrit O.N., Orevi M. (2016). Diagnostic Challenges of Kidney Cancer: A Systematic Review of the Role of Positron Emission Tomography-Computerized Tomography. J. Urol..

[56] Divgi C.R., Uzzo R.G., Gatsonis C., Bartz R., Treutner S., Yu J.Q., Chen D., Carrasquillo J.A., Larson S., Bevan P. (2013). Positron emission tomography/computed tomography identification of clear cell renal cell carcinoma: Results from the REDECT trial. J. Clin. Oncol..

[57] Kang S.K., Zhang A., Pandharipande P.V., Chandarana H., Braithwaite R.S., Littenberg B. (2015). DWI for Renal Mass Characterization: Systematic Review and Meta-Analysis of Diagnostic Test Performance. Am. J. Roentgenol..

[58] Battagli C., Uzzo R.G., Dulaimi E., Ibanez de Caceres I., Krassenstein R., Al-Saleem T., Greenberg R.E., Cairns P. (2003). Promoter hypermethylation of tumor suppressor genes in urine from kidney cancer patients. Cancer Res..

[59] Costa V.L., Henrique R., Danielsen S.A., Eknaes M., Patrício P., Morais A., Oliveira J., Lothe R.A., Teixeira M.R., Lind G.E. (2011). TCF21 and PCDH17 methylation: An innovative panel of biomarkers for a simultaneous detection of urological cancers. Epigenetics.

[60] de Martino M., Klatte T., Haitel A., Marberger M. (2012). Serum cell-free DNA in renal cell carcinoma: A diagnostic and prognostic marker. Cancer.

[61] Hauser S., Zahalka T., Fechner G., Müller S.C., Ellinger J. (2013). Serum DNA hypermethylation in patients with kidney cancer: Results of a prospective study. Anticancer Res..

[62] Hoque M.O., Begum S., Topaloglu O., Jeronimo C., Mambo E., Westra W.H., Califano J.A., Sidransky D. (2004). Quantitative detection of promoter hypermethylation of multiple genes in the tumor, urine, and serum DNA of patients with renal cancer. Cancer Res..

[63] Nuzzo P.V., Berchuck J.E., Korthauer K., Spisak S., Nassar A.H., Abou Alaiwi S., Chakravarthy A., Shen S.Y., Bakouny Z., Boccardo F. (2020). Detection of renal cell carcinoma using plasma and urine cell-free DNA methylomes. Nat. Med..

[64] Outeiro-Pinho G., Barros-Silva D., Aznar E., Sousa A.I., Vieira-Coimbra M., Oliveira J., Gonçalves C.S., Costa B.M., Junker K., Henrique R. (2020). MicroRNA-30a-5p(me): A novel diagnostic and prognostic biomarker for clear cell renal cell carcinoma in tissue and urine samples. J. Exp. Clin. Cancer Res..

[65] Skrypkina I., Tsyba L., Onyshchenko K., Morderer D., Kashparova O., Nikolaienko O., Panasenko G., Vozianov S., Romanenko A., Rynditch A. (2016). Concentration and Methylation of Cell-Free DNA from Blood Plasma as Diagnostic Markers of Renal Cancer. Dis. Markers.

[66] Urakami S., Shiina H., Enokida H., Hirata H., Kawamoto K., Kawakami T., Kikuno N., Tanaka Y., Majid S., Nakagawa M. (2006). Wnt antagonist family genes as biomarkers for diagnosis, staging, and prognosis of renal cell carcinoma using tumor and serum DNA. Clin. Cancer Res..

[67] Xin J., Xu R., Lin S., Xin M., Cai W., Zhou J., Fu C., Zhen G., Lai J., Li Y. (2016). Clinical potential of TCF21 methylation in the diagnosis of renal cell carcinoma. Oncol. Lett..

[68] He Z.H., Qin X.H., Zhang X.L., Yi J.W., Han J.Y. (2018). Long noncoding RNA GIHCG is a potential diagnostic and prognostic biomarker and therapeutic target for renal cell carcinoma. Eur. Rev. Med. Pharmacol. Sci..

[69] Wu Y., Wang Y.Q., Weng W.W., Zhang Q.Y., Yang X.Q., Gan H.L., Yang Y.S., Zhang P.P., Sun M.H., Xu M.D. (2016). A serum-circulating long noncoding RNA signature can discriminate between patients with clear cell renal cell carcinoma and healthy controls. Oncogenesis.

[70] Xie J., Zhong Y., Chen R., Li G., Luo Y., Yang J., Sun Z., Liu Y., Liu P., Wang N. (2020). Serum long non-coding RNA LINC00887 as a potential biomarker for diagnosis of renal cell carcinoma. FEBS Open Bio.

