# Supplementary material for: LiKidMiRs: A ddPCR-Based Panel of 4 Circulating miRNAs for Detection of Renal Cell Carcinoma
Source: Cancers (Basel). 2022 Feb 9;14(4):858. doi: 10.3390/cancers14040858 (PMC8869982; doi:10.3390/cancers14040858)
Supplement: Supplementary file 1 [file cancers-14-00858-s001.zip › cancers-1589496-supplementary.pdf]

Table S1 – Performance of miRNAs as biomarkers for detection of Renal Cell Tumors.

miRNAs	SE %	SP %	PPV %	NPV %	Accuracy %
hsa-miR-21-5p	61.15	64.06	78.70	43.16	62.07
hsa-miR-155-5p	37.41	92.19	91.23	40.41	54.68
hsa-miR-21-5p/ hsa-miR-155-5p	87.05	56.25	81.21	66.67	77.34
Multiple ROC Curve (hsa-miR-21-5p/ hsa-miR-155-5p)	82.50	49.29	58.24	78.16	71.85

Abbreviations: SE-Sensitivity; SP-Specificity PPV – Positive Predictive Value; NPV – Negative Predictive Value

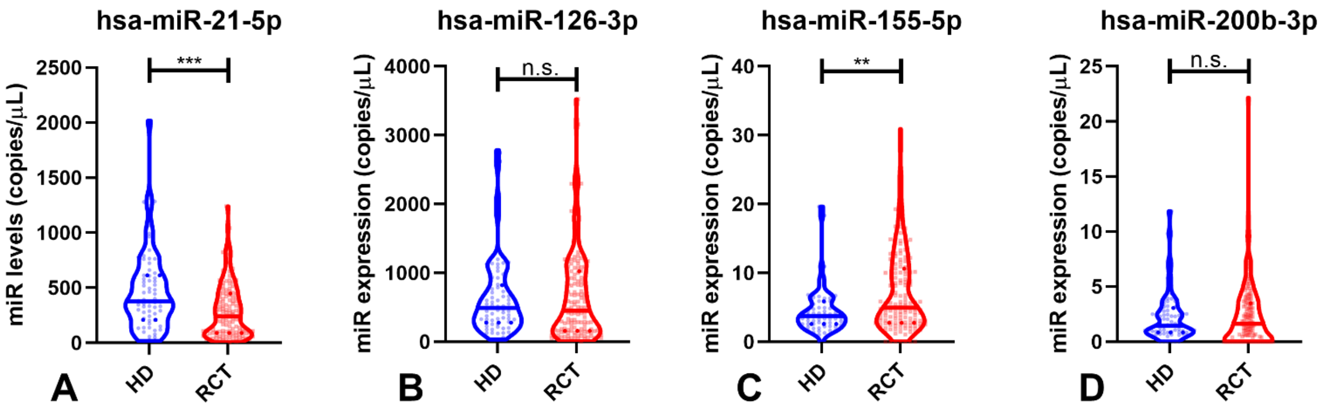

Figure S1 – Violin plots with all points of miRNAs levels in Healthy Donors (HD) and Renal Cell Tumors (RCT) samples of hsa-miR-21-5p (A), hsa-miR-126-3p (B), hsa-miR-155-5p (C) and hsa-miR-200b-3p (D). Dashed lines indicate the interquartile range and horizontal line the median of miR levels. Abbreviations: HD – Healthy Donors; RCT – Renal Cell Tumors; n.s. – not significant, ** – *p*-value < 0.001, *** – *p*-value < 0.0001.

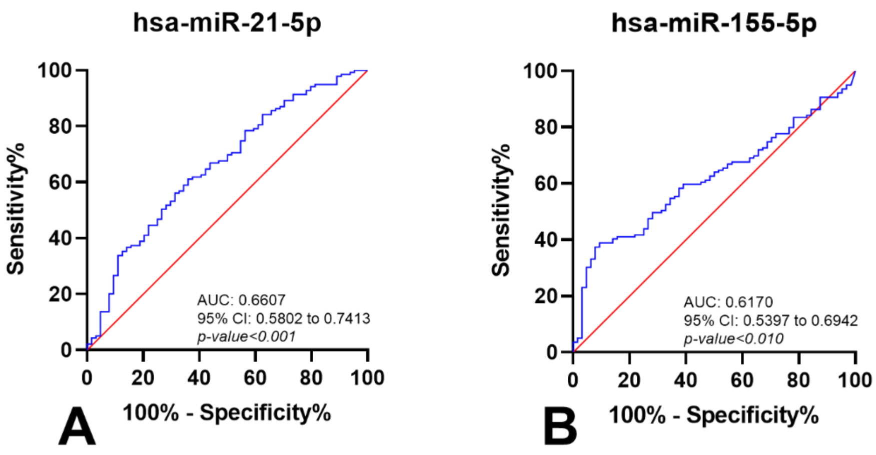

Figure S2 – Receiver Operating Characteristic Curve (without resampling analysis) of hsa-miR-21-5p (A) and hsa-miR-155-5p (B). Red line indicates the reference line and blue line the identity line for each miRNA. Abbreviations: AUC – Area Under the Curve; CI – Confidence Interval.
